# Animal and cellular models of microphthalmia

**DOI:** 10.1177/2633004021997447

**Published:** 2021-02-27

**Authors:** Philippa Harding, Dulce Lima Cunha, Mariya Moosajee

**Affiliations:** UCL Institute of Ophthalmology, London, UK; UCL Institute of Ophthalmology, London, UK; UCL Institute of Ophthalmology, 11-43 Bath Street, London, EC1V 9EL, UK; Moorfields Eye Hospital NHS Foundation Trust, London, UK; Great Ormond Street Hospital for Children NHS Foundation Trust, London, UK; The Francis Crick Institute, London, UK

**Keywords:** cells, development, eye, human, iPSC, microphthalmia, mouse, optic vesicles, organoids, *Xenopus*, zebrafish

## Abstract

Microphthalmia is a rare developmental eye disorder affecting 1 in 7000 births. It is defined as a small (axial length ⩾2 standard deviations below the age-adjusted mean) underdeveloped eye, caused by disruption of ocular development through genetic or environmental factors in the first trimester of pregnancy. Clinical phenotypic heterogeneity exists amongst patients with varying levels of severity, and associated ocular and systemic features. Up to 11% of blind children are reported to have microphthalmia, yet currently no treatments are available. By identifying the aetiology of microphthalmia and understanding how the mechanisms of eye development are disrupted, we can gain a better understanding of the pathogenesis. Animal models, mainly mouse, zebrafish and *Xenopus*, have provided extensive information on the genetic regulation of oculogenesis, and how perturbation of these pathways leads to microphthalmia. However, differences exist between species, hence cellular models, such as patient-derived induced pluripotent stem cell (iPSC) optic vesicles, are now being used to provide greater insights into the human disease process. Progress in 3D cellular modelling techniques has enhanced the ability of researchers to study interactions of different cell types during eye development. Through improved molecular knowledge of microphthalmia, preventative or postnatal therapies may be developed, together with establishing genotype–phenotype correlations in order to provide patients with the appropriate prognosis, multidisciplinary care and informed genetic counselling. This review summarises some key discoveries from animal and cellular models of microphthalmia and discusses how innovative new models can be used to further our understanding in the future.

**Plain language summary:**

**Animal and Cellular Models of the Eye Disorder, Microphthalmia (Small Eye)**

Microphthalmia, meaning a small, underdeveloped eye, is a rare disorder that children are born with. Genetic changes or variations in the environment during the first 3 months of pregnancy can disrupt early development of the eye, resulting in microphthalmia. Up to 11% of blind children have microphthalmia, yet currently no treatments are available. By understanding the genes necessary for eye development, we can determine how disruption by genetic changes or environmental factors can cause this condition. This helps us understand why microphthalmia occurs, and ensure patients are provided with the appropriate clinical care and genetic counselling advice. Additionally, by understanding the causes of microphthalmia, researchers can develop treatments to prevent or reduce the severity of this condition. Animal models, particularly mice, zebrafish and frogs, which can also develop small eyes due to the same genetic/environmental changes, have helped us understand the genes which are important for eye development and can cause birth eye defects when disrupted. Studying a patient’s own cells grown in the laboratory can further help researchers understand how changes in genes affect their function. Both animal and cellular models can be used to develop and test new drugs, which could provide treatment options for patients living with microphthalmia. This review summarises the key discoveries from animal and cellular models of microphthalmia and discusses how innovative new models can be used to further our understanding in the future.

## Introduction

Microphthalmia describes a small underdeveloped eye and is defined as having a total axial length of <19 mm at 1 year of age or <21 mm in an adult measured on B-scan ultrasound, determined as being ⩾2 standard deviations below the age-adjusted mean.^1^ It is a rare condition, with an estimated prevalence of 1 in 7000 live births,^2^ resulting from disrupted eye development between 4–8 weeks gestation either due to genetic or environmental factors.^1,3–5^ Currently no preventative or restorative treatments exist to improve vision.

Prospective UK incidence studies have indicated that environmental causes, such as maternal vitamin A deficiency or alcohol consumption, contribute to approximately 2% of microphthalmia cases.^2,3,6–8^ There are over 90 identified monogenic causes, as well as large chromosomal aberrations.^2,3^ The most common mutations associated with microphthalmia are in transcription factors that control correct gene expression during early eye development, such as *SOX2* and *OTX2* which account for 60% of severe bilateral microphthalmia,^9^ along with *RAX*, *VSX2* and *PAX6.*^2,3^ These transcription factors regulate signalling pathways (e.g. WNT, BMP, TGFβ and SHH) which stimulate morphogenic movements and specialisation of cells within the developing eye. Retinoic acid signalling is vital for early eye morphogenesis, and functional variants in this pathway frequently cause microphthalmia, including *STRA6*, *ALDH1A3*, *RARβ* and *RBP4.*^2,3^ Inheritance patterns comprise autosomal dominant, recessive and X-linked, although germline mosaicism has been reported for multiple microphthalmia-associated variants, making deciphering inheritance patterns and providing appropriate genetic counselling challenging.^3,10–14^ Most pathogenic mutations associated with non-syndromic cases arise *de novo* sporadically, and include missense, nonsense, frameshift and splice-site variants.^2,3,15^ A molecular diagnosis can be obtained in approximately 70% of severe bilateral microphthalmia cases, but less than 10% of unilateral cases, which consists of the majority of patients.^2,3,16^ This discrepancy may be the result of *de novo* mutations, mosaicism and haploinsufficiency in unilateral patients, or due to genetic/epigenetic modifiers, but has not been thoroughly investigated.^17–19^

Heterogeneity in clinical phenotype is observed amongst patients. Microphthalmia can manifest as an isolated condition with a continuum of severity and laterality, often in association with anophthalmia (in the contralateral eye) or ocular coloboma (in the same or contralateral eye), which are considered part of the same spectrum of ocular disorders (collectively known as MAC). An affected eye can display other ocular features (complex) such as cataract, anterior segment dysgenesis or retinal dystrophy, and 33–95% of patients exhibit systemic features (syndromic) (detailed by gene in Harding and Moosajee 2019).^20–22^ Variable expressivity and non-penetrance have also been observed in microphthalmia probands.^3,12,23^ The severity of microphthalmia on visual function depends on the stage in which eye development was disrupted, and so the degree to which ocular structure and cellular function is perturbed, as well as associated ocular malformations present.

Identification of causal microphthalmic genes and the pathways disrupted in eye development will provide insight into pathogenesis as well as potential therapeutic targets to treat infants born with microphthalmia, through ocular delivery of drug compounds to stimulate postnatal growth and development.^3^ Furthermore, uncovering environmental factors and genetic modifiers is key to begin understanding variable penetrance.^24,25^ By recognising the effects of specific variants on clinical phenotype, we can establish genotype–phenotype correlations, provide important prognostic indicators and allow assembly of the correct multidisciplinary team and for families to receive informed genetic counselling and access to family planning advice.

As microphthalmia arises within the first few weeks of gestation, it is difficult to study eye development in humans, both morphologically and molecularly. Consequently, much of our understanding of microphthalmia derives from animal and cellular models.^26–29^ This review highlights the key disease models used to study microphthalmia, as well as the innovative technologies which will further our understanding and aid the generation of pioneering treatments.

## Eye development and microphthalmia

Eye formation begins relatively early in vertebrate embryogenesis, from 3 weeks gestation in humans, embryonic day 8 (E8.0) in mice, 12 h post fertilisation (hpf) in zebrafish and embryonic stage 12.5 in *Xenopus* (Table 1). The molecular mechanisms of early eye development and the pathways relating to microphthalmia are reviewed in detail by Harding and Moosajee.^3^ Briefly, the eye is initially specified in the anterior neural plate through the upregulation of eye field transcription factors (EFTFs), including *RAX*, *PAX6* and *SIX3*, which form a self-regulating network of genes coordinating eye development.^30–33^ The eye field then splits in two as the cells migrate anteriorly away from the midline of the neural plate, evaginating towards the surface ectoderm.^30–32^ Concurrently, the lens develops from a pre-placodal region within the surface ectoderm, and signalling from the evaginating optic vesicle stimulates the thickening of the lens placode, which then reciprocally induces invagination of the optic vesicle to form a bilayered optic cup.^30,34^ The outer layer of the optic cup becomes the retinal pigmented epithelium (RPE), while the inner layer forms the presumptive neural retina (NR), which later differentiates into the specialised cell types of the retina.^32,35^ The opening along the inferior surface of the optic cup (the optic fissure), which allows the hyaloid vasculature to support eye development, closes by week 7 in humans (Table 1).^26^

**Table 1.** Stages of early eye development in humans and animal models.

Species	EFTF expression	Splitting of eye field	Optic vesicle evagination	Optic cup invagination	Closure of the optic fissure	References
Human	22 days	22 days	27 days	28–35 days	35–49 days	Harding and Moosajee^3^; Richardson *et al.*^26^; Patel and Sowden^36^
Mouse	E8.0	E8.5	E9.0–E9.5	E10–E12.5	E11–E13	Patel and Sowden^36^; Cvekl *et al.*^37^; Graw^38^; Reis and Semina^39^
Zebrafish	12 hpf	12–14 hpf	14 hpf	15–28 hpf	48–56 hpf	Richardson *et al.*^26^; Deml *et al.*^40^; Chhetri *et al.*^41^; Kimmel *et al.*^42^
	6–10 somite stage	10–12 somite stage	12 somite stage	12–15 somite stage	Hatching period (long pec stage)	
*Xenopus*	Stage 12.5–15	Stage 16–18	Stage 18–26	Stage 27–34	Stage 38–46	Zuber^27^; Zuber *et al.*^33^; Ledford *et al.*^43^; Holt^44^; Feldman^45^; Henry *et al.*^46^

Days, days gestation; E, embryonic day; EFTF, eye field transcription factor; hpf, hours post fertilisation; stage, *Xenopus* Nieuwkoop and Faber developmental stage.

## Animal models of microphthalmia

**Figure 1.**
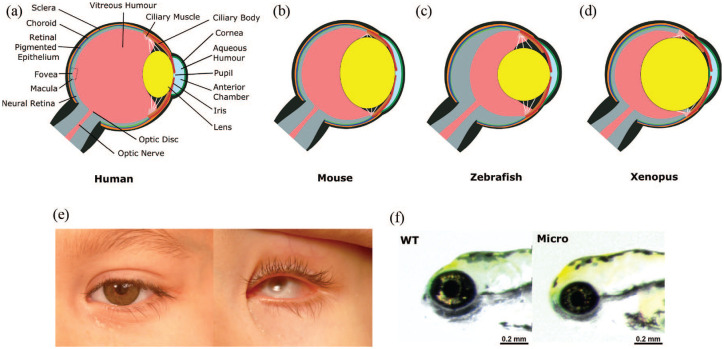
Diagrams of mature eye structure in human, mouse, zebrafish and *Xenopus*, and images of microphthalmic eyes in human and zebrafish. (a) Human eye with a cone-rich macula responsible for central vision, and a small lens which refracts light, along with the cornea. (b) Mouse eye with an enlarged lens compared with humans and lacking a cone-rich macula, with cones instead dispersed throughout the retina. (c) Zebrafish eye with thick neural retina layer and spherical lens which alone is responsible for focusing light. (d) *Xenopus* eye with a large, spherical lens encompassing most of the vitreous. (e) Clinical image of patient with unilateral left microphthalmia with and without prosthetic shell. (f) Wildtype and microphthalmic zebrafish at 76 h post fertilisation (hpf).

**Figure 2.**
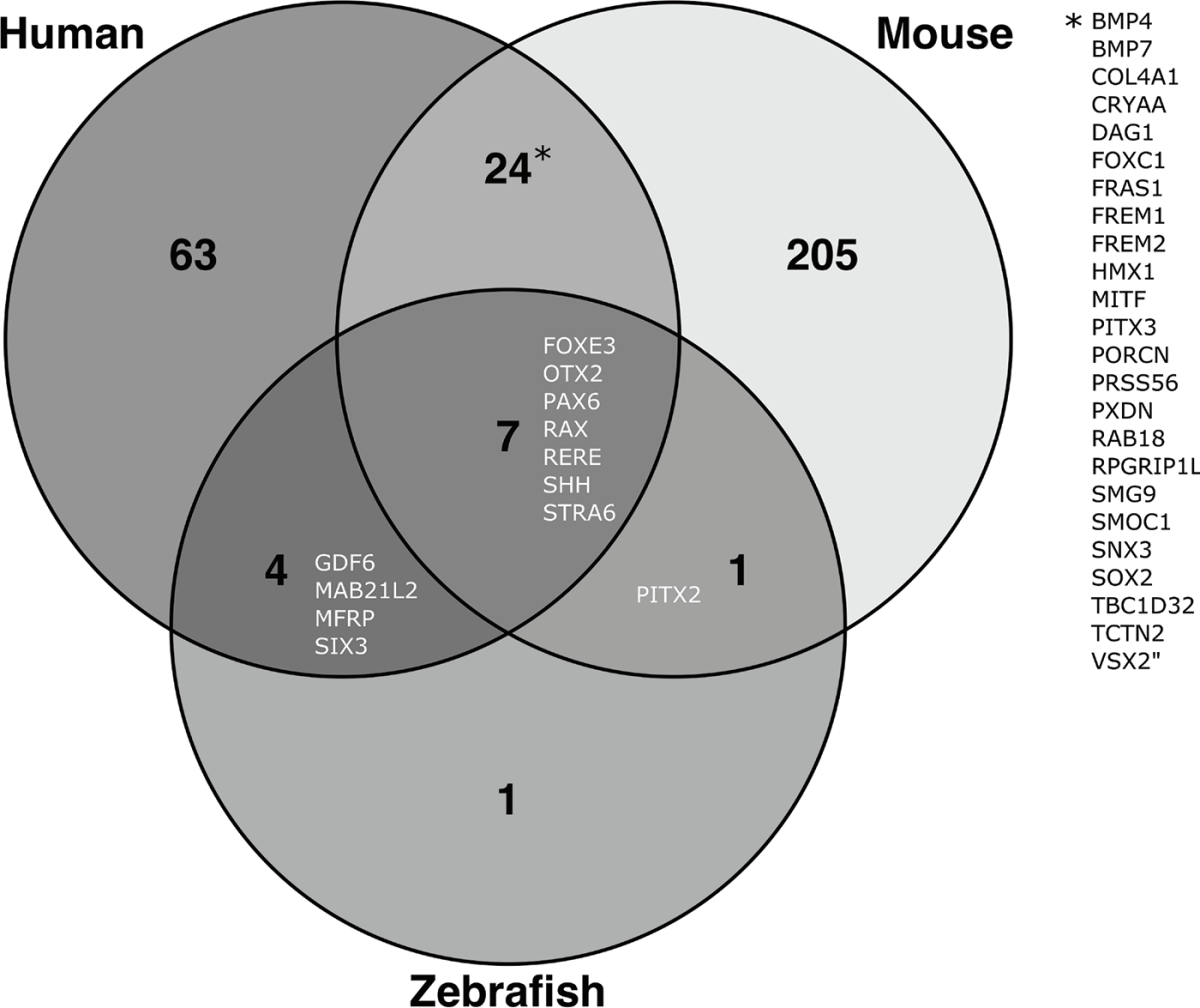
Genes identified to cause microphthalmia in mouse, zebrafish and humans based on database and literature search, with overlapping genes listed. Mouse data from Mouse Genome Informatics database (http://www.informatics.jax.org/). Zebrafish data from Zebrafish Information Network (ZFIN) database (https://zfin.org/). Data from December 2020. Full list of genes in Supplemental Table 1.

Mature eye structure is similar across vertebrate species (Figure 1), with light entering *via* the pupil through the transparent cornea and lens, which refracts the light through ciliary muscle movement, before reaching the stratified, light-sensing NR at the back of the eye (whose function is supported by the RPE) where specialised retinal cells detect light (photoreceptors) and convert it into electrical signals, which are transmitted to the brain *via* the optic nerve. Investigating disease progression and molecular pathways in models with known genetic causes can help understand disease mechanisms. Due to their conserved ocular development and physiology, numerous mouse, zebrafish and *Xenopus* lines with a microphthalmia phenotype have been generated, many of which have overlapping causal molecular changes to patients (Table 2, Figure 2, Table S1). Microphthalmia has been studied in other animal models, including chick^47–49^ and drosophila;^50^ however, this review will only explore findings from mouse, zebrafish and *Xenopus*, as these are the most common models utilised to explore the molecular basis of microphthalmia due to their ease of experimental manipulation together with conserved genetics (Table 3).

**Table 2.** Animal models of known human microphthalmia genes (mouse, zebrafish and *Xenopus*).

Human gene	Animal	Genotype/allele ID	Genotype	Predominant ocular phenotype	Reference(s)
SOX2	Mouse	MGI:3625924	Sox2^tm1Lpev^/Sox2^tm3Lpev^	Mi, An, RD	Taranova *et al.*^51^
		MGI:3625925	Sox2^tm1Lpev^/Sox2^tm4Lpev^	Mi, An, ONH, RD	Taranova *et al.*^51^
OTX2	Mouse	MGI:5573220	Otx2^tm12.1Asim^/Otx2^tm12.1Asim^	Mi, An, ONH, RD	Bernard *et al.*^52^
		MGI:2172552	Otx2^tm1Sia^/Otx2^+^	Mi, An, RD, Ak, AC, ASD, A	Matsuo *et al.*^53^
	Zebrafish	ZDB-ALT-100412-1	otx2b^hu3625^/otx2b^hu3625^	Mi, RD	Bando *et al.*^54^
RAX	Mouse	MGI:5494276	Rax^tm1.1(rTA,tetO-cre)Lan^/?	Mi	Plageman annd Lang^55^
		MGI:3809647	Rax^ey1^/Rax^ey1^	Mi, An, ONH, LS	Chase^56^
	Zebrafish	ZDB-ALT-020514-4	rx3^s399^/rx3^s399^	Mi, An, LS	Yin *et al.*^57^; Loosli *et al.*^58^
VSX2	Mouse	MGI:3799537	Vsx2^or-2J^/Vsx2^or-2J^	Mi, LA	Prochazka *et al.*^59^
		MGI:5449361	Vsx2^or-J^/Vsx2^or-J^	Mi, OHN, RD, LS	Zou and Levine^60^
		MGI:4358055	Vsx2^or^/Vsx2^or^	Mi, ONH, RD, LA	Truslove^61^
		MGI:5449360	Vsx2^tm1.1Eml^/Vsx2^tm1.1Eml^	Mi, RD, LS	Zou and Levine^60^
		MGI:5449358	Vsx2^tm1.1Itl^/Vsx2^tm1.1Itl^	Mi, RD, LS	Zou and Levine^60^
		MGI:5449362	Vsx2^or-J^/Vsx2^tm1.1Eml^	Mi	Zou and Levine^60^
PAX6	Mouse	MGI:2680573	Pax6^1Jrt^/Pax6^+^	Mi, An, RD, LA, CO, IH	Rossant^62^
		MGI:3613473	Pax6^2Neu^/Pax6^+^	Mi, Cat	Favor *et al.*^63^; Favor and Neuhäuser-Klaus^64^
		MGI:3590307	Pax6^3Neu^/Pax6^+^	Mi, Cat	Favor *et al.*^63^; Favor and Neuhäuser-Klaus^64^
		MGI:4943211	Pax6^3Neu^/Pax6^+^	Mi, Cat	Favor *et al.*^65^
		MGI:3590308	Pax6^4Neu^/Pax6^+^	Mi, Cat, IH	Favor *et al.*^63^; Favor and Neuhäuser-Klaus^64^
		MGI:3613474	Pax6^5Neu^/Pax6^+^	Mi, Cat	Favor *et al.*^63^; Favor and Neuhäuser-Klaus^64^
		MGI:3588509	Pax6^6Neu^/Pax6^+^	Mi, Cat	Favor *et al.*^63^; Favor and Neuhäuser-Klaus^64^
		MGI:3613467	Pax6^7Neu^/Pax6^+^	Mi, Cat, IH	Favor *et al.*^63^; Favor and Neuhäuser-Klaus^64^
		MGI:3613475	Pax6^8Neu^/Pax6^+^	Mi	Favor *et al.*^63^
		MGI:3613476	Pax6^9Neu^/Pax6^+^	Mi	Favor *et al.*^63^
		MGI:3613477	Pax6^10Neu^/Pax6^+^	Mi	Favor *et al.*^63^
		MGI:3707321	Pax6^132-14Neu^/Pax6^132-14Neu^	Mi, Col, LA, ASD	Favor *et al.*^66^
		MGI:3611340	Pax6^ADD4802^/Pax6^+^	Mi, LA, Cat, CO	Graw *et al.*^67^
		MGI:5511023	Pax6^Aey80^/Pax6^+^	Mi, LS	Puk *et al.*^68^
		MGI:2175199	Pax6^Coop^/Pax6^+^	Mi, CO, IH	Lyon *et al.*^69^
		MGI:2687018	Pax6^Leca1^/Pax6^Leca1^	Mi, LA	Thaung *et al.*^70^
		MGI:2687019	Pax6^Leca2^/Pax6^Leca2^	Mi, LA	Thaung *et al.*^70^
		MGI:2687020	Pax6^Leca3^/Pax6^Leca3^	Mi, LA	Thaung *et al.*^70^
		MGI:2687021	Pax6^Leca4^/Pax6^Leca4^	Mi, LA	Thaung *et al.*^70^
		MGI:3611468	Pax6^Mhdaaey11^/Pax6^Mhdaaey11^	Mi, Cat, CO	Graw *et al.*^67^
		MGI:3611342	Pax6^Mhdaaey18^/Pax6^+^	Mi, Cat, CO	Graw *et al.*^67^
		MGI:3526886	Pax6^Mhdaaey18^/Pax6^+^	Mi, Cat	European Mouse Mutant Archive^71^
		MGI:3798480	Pax6^Rgsc20^/Pax6^+^	Mi, Cat	RBCGS Center^72^
		MGI:3798889	Pax6^Rgsc123^/Pax6^+^	Mi, Cat	RBCGS Center^72^
		MGI:3799164	Pax6^Rgsc242^/Pax6^+^	Mi, Cat	RBCGS Center^72^
		MGI:2175204	Pax6^Sey-Dey^/Pax6^+^	Mi, Col, RD, LA, LS, Cat, IH, A,	Theiler *et al.*^73^
		MGI:2175206	Pax6^Sey-H^/Pax6^+^	Mi, Col	Hogan *et al.*^74^
		MGI:2175208	Pax6^Sey-Neu^/Pax6^+^	Mi, LA, ASD, IH	Ramaesh *et al.*^75^
		MGI:3771036	Pax6^Sey^/Pax6^+^	Mi	Hill *et al.*^76^
		MGI:2170872	Pax6^Sey^/Pax6^+^	Mi, ONH, RD, LA, ASD	Hill *et al.*^76^
		MGI:5567085	Pax6^tm1.1Zkoz^/Pax6^tm1.1Zkoz^	Mi, RD, LA	Klimova and Kozmik^77^
		MGI:5317872	Pax6^tm1.2Xzh^/Pax6^tm1.2Xzh^	Mi, Col, LA	Carbe *et al.*^78^
		MGI:4821786	Pax6^tm2Pgr^/Pax6^+^	Mi, OHN, LS, ASD	Kroeber *et al.*^79^
		MGI:4366458	Pax6^tm2Pgr^/Pax6^tm2Pgr^	Mi, LS, LA, Cat	Shaham *et al.*^80^
		MGI:4358211	Pax6^tm2Pgr^/Pax6^tm2Pgr^	Mi, RD, LA, LS, Cat, ASD, IH,	Davis *et al.*^81^
	Zebrafish	ZDB-ALT-980203-1333	pax6b^tq253a^/pax6b^tq253a^ (sri)	Mi, LA, ASD	Kleinjan *et al.*^82^
	Xenopus	–	Pax6^−^/^−^	Mi, RD, Ak	Nakayama *et al.*^83^
		–	Pax6^−^/*	Mi, Cat, CO, A	Nakayama *et al.*^83^
STRA6	Mouse	MGI:5490888	Stra6^tm1Nbg^/Stra6^tm1Nbg^	Mi, RH	Ruiz *et al.*^84^
	Zebrafish	ZDB-ALT-180521-1	stra6l^musc97^/stra6l^musc97^	Mi	Shi *et al.*^85^
FOXE3	Mouse	MGI:2175026	Foxe3^dyl^/Foxe3^dyl^	Mi, LA, LS, Cat, CO, ASD	Sanyal and Hawkins^86^
		MGI:3604813	Foxe3^tm1Mjam^/Foxe3^tm1Mjam^	Mi, RD, ASD, LA,	Medina-Martinez *et al.*^87^
	Zebrafish	ZDB-ALT-181015-1	Foxe3^s4001^/Foxe3^s4001^	Mi, LA, LS	Krall *et al.*^88^
BMP4	Mouse	MGI:3711773	Bmp4^tm1Blh^/Bmp4^+^	Mi, An, ONH, RD, Cat, CO, AC, ASD, IH,	Dunn *et al.*^89^
BMP7	Mouse	MGI:3629218	Bmp7^tm2Rob^/Bmp7^tm4(Bmp4)Rob^	Mi	Zouvelou *et al.*^90^
		MGI:2451062	Bmp7^tm1Rob^/Bmp7^tm1Rob^	Mi, An	Dudley *et al.*^91^
		MGI:3847892	Bmp7^tm1.2Dgra^/Bmp7^tm1.2Dgra^	Mi, An, RD, LA	Oxburgh *et al.*^92^
GDF6	Zebrafish	ZDB-ALT-980203-555	gdf6a^s327^/^s327^ (dark half)	Mi	French *et al.*^93^; Pant *et al.*^94^
		ZDB-ALT-050617-10	gdf6a^m233^/^m233^ (out)	Mi, An	den Hollander *et al.*^95^
SMOC1	Mouse	MGI:4941783	Smoc1^Tn(sb-lacZ,GFP)PV384Jtak^/Smoc1^Tn(sblacZ,GFP)PV384Jtak^	Mi, ONH, RD	Okada *et al.*^96^
SHH	Mouse	MGI:3759227	Shh^tm1Amc^/Shh^tm2Amc^	Mi, ONH, RD	Wang *et al.*^97^; Dakubo *et al.*^98^
		MGI:3812210	Shh^tm1Chg^/Shh^+^	Mi, An	Ratzka *et al.*^99^
		MGI:3589447	Shh^tm1Chg^/Shh^+^	Mi, Ak	Grobe *et al.*^100^
		MGI:3042780	Shh^tm1Chg^/Shh^tm1Chg^	Mi	Bulgakov *et al.*^101^
		MGI:3851497	Shh^tm1.1Rseg^/Shh^tm1.1Rseg^	Mi	Chan *et al.*^102^
		MGI:3851498	Shh^tm1Amc^/Shh^tm1.1Rseg^	Mi	Chan *et al.*^102^
	Zebrafish	ZDB-ALT-980413-636	shha^tq252^/shha^tq252^ (syu)	Mi, RD	Brand *et al.*^103^; Stenkamp *et al.*^104^
MAB21L2	Zebrafish	ZDB-ALT-140130-18	mab21l2 ^au10^/mab21l2 ^au10^	Mi, Col, LA, ASD	Gath and Gross^105^
		ZDB-ALT-150611-1	mab21l2^Q48Sfs*5^/mab21l2^Q48Sfs*5^	Mi, Col, CO	Deml *et al.*^40^
		ZDB-ALT-150611-2	mab21l2^R51_F52del^/mab21l2^R51_F52del^	Mi, An, Col, RD, ASD	Deml *et al.*^40^
PORCN	Mouse	MGI:6368187	Porcn^tm1.1Lcm^/Porcn^+^	Mi, Col, RD	Bankhead *et al.*^106^
FRAS1	Mouse	MGI:2657302	Fras1^bl^/Fras1^bl^	Mi	Phillips^107^
FREM1	Mouse	MGI:3026630	Frem1^crf11^/Frem1^crf11^	Mi	Kile *et al.*^108^; Beck *et al.*^109^
		MGI:5473606	Frem1^eyes2^/Frem1^eyes2^	Mi	Beck *et al.*^110^
TCTN2	Mouse	MGI:5292219	Tctn2^tm1.1Reit^/Tctn2^tm1.1Reit^	Mi	Sang *et al.*^111^
COL4A1	Mouse	MGI:4822250	Col4a1^D456^/Col4a1^+^	Mi, LA, Cat	Favor *et al.*^112^
		MGI:5308056	Col4a1^deltaex40^/Col4a1^+^	Mi, ONH, RD	Labelle-Dumais *et al.*^113^
		MGI:4822242	Col4a1^ENU911^/Col4a1^+^	Mi, LA, Cat, CO	Favor *et al.*^112^
PTCH2	Zebrafish	–	ptch2^uta4^/ptch2^uta4^	Mi, LA, Cat, ASD	Lee *et al.*^114^
		–	ptch2^uta5^/ptch2^uta5^	Mi, RD	Lee *et al.*^114^
		–	ptch2^uta6^/ptch2^uta6^	Mi, RD	Lee *et al.*^114^
		–	ptch2^uta16^/ptch2^uta16^	Mi, LA	Lee *et al.*^114^
		–	ptch2^uta17^/ptch2^uta17^	Mi	Lee *et al.*^114^
		–	ptch2^uta19^/ptch2^uta19^	Mi, Cat	Lee *et al.*^114^
		–	ptch2^uta20^/ptch2^uta20^	Mi, Cat	Lee *et al.*^114^
		–	ptch2^uta22^/ptch2^uta22^	Mi, Cat	Lee *et al.*^114^
TBC1D32	Mouse	MGI:5560506	Tbc1d32^b2b2596Clo^/Tbc1d32^b2b2596Clo^	Mi, An	Lo^115^
MFRP	Zebrafish	ZDB-ALT-180816-9	Mfrp^mw78^/mfrp^mw78^	Mi, RD	Collery *et al.*^116^
PRSS56	Mouse	MGI:5444191	Prss56^glcr4^/Prss56^glcr4^	Mi, ONH, RD, ASD	Nair *et al.*^117^
PXDN	Mouse	MGI:5584292	Pxdn^mhdakta048^/Pxdn^mhdakta048^	Mi, ONH, RD, LA, ASD, IH	Yan *et al.*^118^
PITX2	Mouse	MGI:1857844	Pitx2^tm1Sac^/Pitx2^+^	Mi, Cat	Gage *et al.*^119^
		MGI:1857846	Pitx2^tm2Sac^/Pitx2^tm2Sac^	Mi, LA	Gage *et al.*^119^
		MGI:2445429	Pitx2^tm4(cre)Jfm^/Pitx2^+^	Mi, LA, LS, CO, IH	Liu and Johnson^120^
	Zebrafish	ZDB-ALT-180731-2	pitx2^M64*^/pitx2^M64*^	Mi, An, ASD, IH	Hendee *et al.*^121^
PITX3	Mouse	MGI:4429423	Pitx3^eyl^/Pitx3^eyl^	Mi, RD, Ak	Rosemann *et al.*^122^
		MGI:3042029	Pitx3^ak^/Pitx3^ak^	Mi, RD, LA, ASD, IH	Varnum and Stevens^123^
MITF	Mouse	MGI:2662939	Mitf^Mi-Crc^/Mitf^Mi-Crc^	Mi	Hetherington^124^
		MGI:3525852	Mitf^mi-ce^/Mitf^mi-ce^	Mi, RD, LA, Cat	Zimring *et al.*^125^
		MGI:4455018	Mitf^Mi^/Mitf^Mi^	Mi	Steingrímsson *et al.*^126^
		MGI:2663064	Mitf^Mi-wh^/Mitf^mi-x^	Mi	Munford^127^
		MGI:4442409	Mitf^mi-x39^/Mitf^mi-x39^	Mi	Hallsson *et al.*^128^
		MGI:3630349	Mitf^Mi-ws^/Mitf^Mi-ws^	Mi, RD	Hollander^129^
		MGI:3762342	Mitf^Mi-wh^/Mitf^Mi-wh^	Mi, Col, ONH, RD	Packer *et al.*^130^
		MGI:4455017	Mitfmi-^vga9^/Mitf^mi-vga9^	Mi, RD	Steingrímsson *et al.*^126^
		MGI:4356490	Mitf^mi-tg^/Mitf^mi-tg^	Mi, IH	Krakowsky *et al.*^131^
		MGI:4410320	Mitf^mi-rw^/Mitf^mi-rw^	Mi, RD	Southard^132^
		MGI:4356528	Mitf^Mi-Or^/Mitf^Mi-Or^	Mi, An, RD	Steingrímsson *et al.*^126^; Stelzner ^133^
		MGI:3041536	Mitf^mi-Mhdabcc2^/Mitf^mi-Mhdabcc2^	Mi	Hansdottir *et al.*^134^
		MGI:5307227	Mitf^Mi^/Mitf^Mi-J^	Mi, RD	Silvers *et al.*^135^
		MGI:4455020	Mitf^mi-ew^/Mitf^mi-ew^	Mi	Steingrímsson *et al.*^126^
		MGI:4442432	Mitf^mi-enu198^/Mitf^mi-enu198^	Mi	Hallsson *et al.*^128^
		MGI:3587635	Mitf^mi-enu122^/Mitf^mi-enu122^	Mi, RD	Steingrímsson *et al.*^126^
		MGI:3041533	Mitf^mi-enu5^/Mitf^mi-enu5^	Mi	Hansdottir *et al.*^134^
		MGI:3522321	Mitf^mi-di^/Mitf^mi-di^	Mi, RD	West *et al.*^136^
FOXC1	Mouse	MGI:3802472	Foxc1^hith^/Foxc1^hith^	Mi, LA, ASD, IH	Zarbalis *et al.*^137^
CRYAA	Mouse	MGI:3690118	Cryaa^2J^/Cryaa^2J^	Mi, LS, Cat	Xia *et al.*^138^
		MGI:2653233	Cryaa^Aey7^/Cryaa^Aey7^	Mi, LA, Cat	Graw *et al.*^139^
		MGI:3784583	Cryaa^tm1.1Ady^/Cryaa^tm1.1Ady^	Mi, LA, LS, Cat	Xi *et al.*^140^
		MGI:2175799	Cryaa^tm1Wawr^/Cryaa^tm1Wawr^	Mi, LS, Cat	Brady *et al.*^141^
		MGI:2653234	Cryaa^Aey7^/Cryaa^+^	Mi, LA, Cat	Graw *et al.*^139^
FREM2	Mouse	MGI:5618921	Frem2^ne^/Frem2^ne^	Mi	Lo^115^
		MGI:3603819	Frem2^my-F11^/Frem2^my-F11^	Mi, An	Timmer *et al.*^142^
		MGI:3796628	Frem2^b2b3270Clo^/Frem2^b2b3270Clo^	Mi, An	Curtain and Donahue^143^
RPGRIP1L	Mouse	MGI:3716631	Rpgrip1l^tm1Urt^/Rpgrip1l^tm1Urt^	Mi	Vierkotten *et al.*^144^; Delous *et al.*^145^
SMG9	Mouse	MGI:5776357	Smg9^em1(IMPC)J^/Smg9^em1(IMPC)J^	Mi	Shaheen *et al.*^146^
SIX3	Zebrafish	ZDB-ALT-160421-3, ZDB-ALT-071211-1	six3a^vu129^/six3a^vu129^, six3b^vu87^/six3b^vu87^	Mi, RD	Samuel *et al.*^147^
SNX3	Mouse	MGI:5767809	Snx3^tm1.1(KOMP)Vlcg^/Snx3^tm1.1(KOMP)Vlcg^	Mi, An	Mouse Genome Informatics and the International Mouse Phenotyping Consortium^148^
DAG1	Mouse	MGI:4440460	Dag1^tm2Kcam^/Dag1^tm2Kcam^	Mi, Bu, CO	Satz *et al.*^149^
HMX1	Mouse	MGI:3838401	Hmx1^dmbo^/Hmx1^dmbo^	Mi	Munroe *et al.*^150^
RERE	Mouse	MGI:3577769	Rere^eyes3^/Rere^eyes3^	Mi	Kim *et al.*^151^
		MGI:5503952	Rere^eyes3^/Rere^om^	Mi	
	Zebrafish	ZDB-ALT-980203-1102, ZDB-ALT-980203-311	rerea^tb210^/rerea^tw220c^	Mi, ONH, RD	Plaster *et al.*^152^; Schilling *et al.*^153^
RAB18	Mouse	MGI:5698703	Rab18^m1Hongc^/Rab18^m1Hongc^	Mi, ONH	Cheng *et al.*^154^

Mouse genotype ID and phenotypic data was taken from the Mouse Genome Informatics database (http://www.informatics.jax.org/). Zebrafish allele ID was taken from The Zebrafish Information Network (ZFIN) database (https://zfin.org/). *Xenopus* data was taken from Xenbase (http://www.xenbase.org). Data from December 2020.

A, aniridia; AC, absent cornea; Ak, aphakia; An, anophthalmia; ASD, anterior segment dysgenesis; Bu, buphthalmos; Cat, cataract; CO, corneal opacity; Col, coloboma; IH, iris hypoplasia; LA, lens abnormalities; LS, small lens; Mi, microphthalmia; ONH, optic nerve hypoplasia; RD, retina dysplasia.

**Table 3.** Advantages and disadvantages of mouse, zebrafish, *Xenopus* and 2D/3D cellular models of microphthalmia.

Model	Size of ocular structure	Availability of material	Cost	Time to develop mature ocular structure	Genetic conservation with humans	Morphological similarity to humans	Availability of genetic/phenotypic data
Mouse	Large (3 mm)	Breed in medium numbers (5–10 pups per litter)	High	1–2 months	High	High	Very good
Zebrafish	Medium (1–2 mm)	Breed in large numbers (>100 fertilised eggs/clutch)	Medium	3–5 days	Low	High	Good
*Xenopus*	Large (3–6 mm)	Breed in large numbers (>100 fertilised eggs/clutch)	Medium	3–5 days	Medium	High	Poor
Cellular – 2D	N/A	Easy to expand (although can be difficult to obtain primary patient/embryonic tissue)	Medium	N/A	Human	N/A	Very good
Cellular – 3D	Small (100–500 µm)	Protocols to obtain mature structures can be inefficient (and difficulty obtaining primary patient/embryonic tissue)	High	2–6 months (plus 2–3 months to reprogramme primary cells to iPSCs if required)	Human	Medium (mature structure does not contain vasculature etc)	Very good

### Mouse

#### Advantages of mouse models of ocular development

Mice are the most common animal model for studying development and disease.^155,156^ They are easy to manage in a laboratory environment, being small with a short generation time, and are relatively cost effective.^157^ The mouse is the best characterised mammalian model system for hereditary disease, and 99% of its genome is conserved compared with humans.^25,158^ Eye development between humans and mice is similar (Table 1), and the mature ocular structure resembles the human, albeit lacking the cone-rich macula, instead with few cone photoreceptor cells distributed evenly throughout the retina (Figure 1).^159^ The size of the mouse eye permits morphological analysis without the need for advanced technical equipment.^25,38^

#### Generation of microphthalmic mice

Historically, forward genetics was used to create phenotypes randomly in animals, and those of interest were screened to identify genetic mutations.^25,156^ However, recent advances in genome editing technologies means it is now more common to directly modify specific genes to create mouse models using targeted or conditional mutagenesis, thereby using reverse genetics to generate specific mutants.^25,156^ Mouse phenotyping centres, such as the International Mouse Phenotyping Consortium (IMPC, https://www.mousephenotype.org/) are used to screen targeted mutants for ocular phenotypes.^25,155^ Many mutant mouse lines have been generated (Table 2), and 269 genes or loci have been linked to microphthalmia, from which key discoveries have been made and are described in Graw^25^ (Figure 2, Table S1).

#### Drawbacks of mouse ocular models

Despite the shared genetics of mice and humans, discrepancy in ocular phenotypes implies divergence in molecular regulatory mechanisms between the species. For example, common microphthalmia-associated genes involved in retinoic acid signalling, such as *ALDH1A3* and *RARβ*, do not produce a microphthalmic phenotype in mouse models, which instead suffer from ocular disorders including lens and retinal anomalies.^3,25,160,161^ On the other hand, mutations in some genes produce a more severe ocular phenotype in mice than typically observed in humans, such as *Pax6* which was first studied as a classical anophthalmia model as many mutants display no eyes, while microphthalmic models often develop many additional eye defects (Table 2).^25^ A further problem with mouse models is that inbred strains can be associated with background ocular disorders; for example, 5–10% of C57BL/6 mice and related strains develop sporadic microphthalmia/anophthalmia, depending on age, environment and additional induced mutations, most likely as the result of a polygenic disease basis.^156^ Therefore, choice of strain is important for translating results in mice to understanding human eye development and disease, as well as when testing novel therapies.

#### Understanding molecular pathways in microphthalmia

Conservation of genetics makes mice a practical model for investigation of genetic pathways in microphthalmia development. Through transcriptome and proteome analysis of knockout mouse models, downstream targets of disease-causing genes can be identified to resolve complex molecular networks. Transcription factor *Pitx3* is known to regulate a large number of molecular elements which are important for eye development.^25^ Homozygous deletion of the promoter region of *Pitx3* or homozygous nonsense mutations resulting in overexpression of truncated protein lead to severe microphthalmia and aphakia due to halted lens development.^123,162–164^ Investigation of molecular targets through EMSA and ChIP assays demonstrated *Pitx3* binds to an evolutionarily conserved region of *Foxe3*, resulting in increased transcriptional activity.^165^ *Foxe3* mutants display a similar phenotype to *Pitx3* including microphthalmia and aphakia (Table 2), reflecting the phenotypic similarity of patients with pathogenic mutations in *PITX3* causing anterior segment dysgenesis 2 (OMIM #610256) and *FOXE3* producing Cataract 11 (OMIM #610623), both of which result in microphthalmia, cataract, anterior segment disorders and sclerocornea, indicating conservation of molecular regulatory mechanisms.^16,166^ Understanding the interaction and shared pathways of genes in eye development helps to clarify genotype–phenotype correlations in patients, as well as identify potentially effective therapeutic targets.

#### Modelling variable ocular and syndromic phenotypes

One of the earliest mouse models of microphthalmia was an ‘eyeless’ mouse (*Rax*^ey^ – MGI: 3809647) in which 10% of mice were reported to develop ‘small’ or ‘medium’ sized eyes, later determined to be the result of a point mutation in transcriptional regulator *Rax*, creating a hypomorphic mutant protein with a partial loss-of-function.^25,56^ Variation in eye size observed in patient cohorts and animal models [like *Rax* and *Pax6* mutant mice (Table 2)] is the result of microphthalmia and anophthalmia (no eye) sharing the same clinical spectrum and genetic basis, which may be the result of dose-dependent gene function.^3^ The availability of allelic series of mouse mutants with a wide range of ocular disorders makes them an ideal model to study the effect of gene dosage on eye development (Table 2). For example, *Sox2* mutants with a range of pathogenic variants display a spectrum of disease severity which correlates to the expression level of *Sox2* in the progenitor cells of the NR, validating a role of dosage sensitivity in microphthalmia (Table 2).^51^ *Mitf* variants have differing effects on gene function: (i) semi-dominant mutations affecting the DNA-binding or transcriptional activation domains yield proteins which do not bind to DNA but still dimerise to other proteins, thereby impairing their functional DNA-binding ability; (ii) recessive variants affect *Mitf* transcription or produce mutant proteins which do not dimerise, and hence do not interfere with DNA binding of other proteins.^167^ This results in a variable ocular phenotype between heterozygotes, homozygotes and compound heterozygotes (Table 2). Similarly, patients with biallelic *MITF* pathogenic mutations exhibit COMMAD (coloboma, osteopetrosis, microphthalmia, macrocephaly, albinism, and deafness) syndrome (OMIM #617306), but haploinsufficient heterozygotes display more mild symptoms of Waardenburg syndrome (OMIM #193510), and patients with semi-dominant heterozygous mutations have the more severe overlapping disorder Tietz albinism-deafness syndrome (OMIM #103500), neither of which include microphthalmia.^167^ Consequently multiple models are required to understand the full spectrum of ocular and systemic features which can be caused by disruption of an individual gene.^25^

Beyond the ocular phenotype, mutant mice with comprehensive phenotype annotation can be used to study any systemic involvement associated with candidate genes. For example, *Otx2*^+/−^ mice display microphthalmia and otocephaly, alongside reduced fertility in males, reflective of the abnormal development of the hypothalamic–pituitary–gonadal axis seen in humans with *OTX2* mutations causing syndromic microphthalmia 5 (MCOPS5 – OMIM #610125).^53,168–172^ These features coincide as, in addition to controlling oculogenesis, *Otx2* regulates the expression of genes involved in pituitary development, such as *Hesx1.*^168,173,174^ Investigation of extraocular phenotypes in mice can provide information on the effect of different variants and genetic/environmental factors on systemic involvement, thus unlocking genotype–phenotype relationships.

#### Identification of novel variants through mouse studies

Forward genetic approaches and phenotypic screening to generate and catalogue eye phenotypes lead to successful discovery of many disease-causing microphthalmia genes, including *Mitf*, first identified in the early *mi* mouse line, and subsequently in a multitude of different lines with a range of ocular defects including a small eye (Table 2).^25,167,175–178^ Targeted mutagenesis is now performed more frequently, allowing validation and further exploration into novel candidate genes, although it is time-consuming and costly to test the effect of genes of uncertain significance, unless performed by large consortiums such as IMPC.^25,155^

#### Genetic modifiers in microphthalmia aetiology

The vast number of mouse lines with a microphthalmic phenotype means the effect of the genetic background can be investigated by inducing multiple mutations in the same mouse model, or the same mutation in multiple strains. *Cx50*^−/−^ mice have microphthalmia with nuclear cataracts.^179,180^ When studying *Cx50* knockouts in both 129S6 and C57BL/6J strains, genetic modifiers were found to influence cataract severity due to differentially altered solubility of crystallin proteins, while eye growth was unaffected by genetic background.^181^ Understanding oligogenic effects on ocular development using these techniques could aid in understanding the variation of MAC spectrum and additional ocular/extraocular features observed within and between families with the same molecular diagnosis.

#### Modelling environmental causes of microphthalmia in mice

Only 2% of microphthalmia cases were attributed to environmental factors in a UK prospective incidence study; however, this varies in different regions of the world.^2,3,6–8^ These include maternal vitamin A deficiency, *in utero* exposure to toxic/teratogenic substances such as alcohol, and certain infections, for example rubella.^8,182–186^ For decades, mice have been used to study the effect of the maternal environment on eye development on embryos, due to their *in utero* gestation, for example, induction of microphthalmia through maternal exposure to toxic trypan blue at 7 days’ gestation.^187,188^ More recently, a study of maternal diabetes showed embryonic glucose exposure mimicking hyperglycaemia and diminished expression of Wnt-PCP pathway genes, resulting in altered cytoskeletal organisation, cell shape and cell polarity in the optic vesicle and ultimately ocular defects including anophthalmia and microphthalmia.^189^ Likewise, the effect of maternal diet, including folic acid deficiency,^190^ alcohol consumption,^191,192^ pharmaceuticals^193^ and infection^194,195^ has also been explored in relation to mouse eye development. Studies on the environmental influences on ocular development are vital to understanding pathogenesis and providing appropriate clinical guidance and care during pregnancy.

### Zebrafish

#### Advantages of zebrafish models of ocular development

Zebrafish (*Danio rerio*) are a popular organism for studying vertebrate eye development and related disorders.^26,41^ Zebrafish are easily maintained and breed in large numbers at low cost, with a generation time of 2–4 months.^24,26,41^ They have many advantages over other organisms such as mice, including external fertilisation, transparency of embryos permitting direct visualisation of organogenesis, rapid eye development leading to adult-like patternation by 72 hpf and a highly organised heterotypical photoreceptor mosaic, which is cone-rich similar to humans, unlike mice.^24,26,41,196^ Overall, zebrafish eye development closely resembles that of humans, although there are some distinctions. Hollow optic vesicles extend from the forebrain at 27 days’ gestation in humans, while in contrast zebrafish optic vesicles begin as a solid mass of cells, which undergo cavitation by 14 hpf (Table 1).^24,26,197^ The thickening of the highly proliferating NR and thinning of the RPE through cell flattening occurs earlier in zebrafish development, and ultimately a wider NR layer is present in the adult zebrafish with squamous epithelial cells in the RPE, where cells in the mature human RPE maintain a cuboidal shape (Figure 1).^24,41,198,199^ Beyond this, the mature eye is remarkably similar between humans and zebrafish, except that in the fish, like many aquatic vertebrates, the larger, spherical lens is solely responsible for focusing light, without contribution from the cornea (Figure 1).^24,198^

#### Generation of microphthalmic zebrafish

There is significant genetic conservation between humans and zebrafish, with 70% of human genes corresponding to at least one zebrafish orthologue, and 84% of known human disease-causing genes having a zebrafish counterpart, providing potential to model a wide scope of human conditions.^26,200^ Moreover, zebrafish are highly amenable to genetic manipulation, meaning mutations can be induced easily. Injection of genome modification tools at the single-cell stage of the fertilised egg allows induction of genetic changes which display a phenotype in the F0 generation.^24,26^ Knockdown morphants can be generated by injection of an antisense oligonucleotide morpholino, which is complementary to the mRNA of interest, and leads to transient gene knockdown in the embryo for up to 5 days post fertilisation (dpf).^1^ Injection of TALENs or CRISPR/Cas9 gene editing tools can be used to generate specific mutations in models, and establish stable mutant lines, which prevent mosaicism and are important to carry out a more complete investigation of phenotypes.^24,201^

#### Drawbacks of zebrafish ocular models

Due to a whole-genome duplication which occurred in zebrafish ancestry, many orthologues of mammalian genes have two copies. Consequently, careful experimental planning is required when undertaking any genetic manipulation to avoid genetic compensation/redundancy. Role-sharing between multiple orthologous genes can lead to variations in phenotypic severity, e.g. missense mutations in the sunrise (sri) *pax6b* homozygous line replicate the milder microphthalmia phenotype observed in patients with some missense *PAX6* mutations, while morpholino-induced knockdown of *pax6a* shows more extreme phenotypes, including reduced body size and abnormal brain development, in addition to microphthalmia.^26,82,202,203^ Moreover, morpholinos can produce variable phenotypes, particularly regarding eye morphology (often spanning the MAC spectrum), and concerns have been raised with regards to their reliability, given disparities between morpholino and mutant phenotypes.^26,204–206^ However, off-target effects can be controlled for by co-injecting with p53 morpholino to mitigate non-specific phenotypes.^207^

#### Understanding molecular pathways in microphthalmia

The shared molecular basis of human and zebrafish eye development means complex genetic networks underpinning microphthalmia can be resolved using transgenic/mutant zebrafish lines to establish the function of genes during eye development (Table 2). The functional role of the *shh* signalling pathway in retinal cell proliferation and survival was established using *syu* mutants, which have homozygous *shha* deletions causing reduced eye size due to decreased mitosis and increased apoptosis in the retina.^103,104^ *rx3*^−/−^ mutants display an eyeless phenotype and expanded forebrain, similar to isolated microphthalmia 3 (OMIM #611038) in patients with biallelic *RAX* mutations.^57^ Transcriptome analysis of these mutants showed downregulation of transcription factors regulating eye development (such as *mab21l2*) and retinoic acid signalling pathway components (including *aldh1a3*), with upregulation of Wnt signalling pathway components which function in brain development and are associated with microphthalmia and multiple neural disorders. Investigation of *mab21l2* morpholino-induced knockdown showed a similar phenotype to *rx3*^−/−^ mutants, validating the downstream role of *mab21l2* in the *rx3* ocular regulatory network, and its role in microphthalmia development.^208^ Other genetic knockdowns inducing microphthalmia include: transcription factors *otx2*,^39^ *rax*,^209^ *six6*^210,211^ and *alx1*;^212^ retinoic acid signalling components *rarβ*^213^ and *aldh1a3*;^160^ TGFβ signalling component *gdf6*;^214^ and SHH signalling component *ptch1.*^215^ There are few established microphthalmic mutant zebrafish lines (Table 2), and most exist from ENU mutagenesis screens.

#### Modelling variable ocular and syndromic phenotypes

Heterogenous ocular and systemic features observed in patient cohorts are mirrored in genetically modified zebrafish, allowing for further analysis of the sources of phenotype variation, whether genetic, epigenetic or environmental. Functional knockdown using *vsx2* morpholinos shows concentration-dependent reduction in eye size. This dosage effect of *vsx2* may explain the variable MAC phenotype observed in *VSX2* patients with isolated microphthalmia 2 (OMIM #610093) or colobomatous microphthalmia 3 (MCOPCB3 – OMIM #610092).^216^ Loss-of-function biallelic variants in human *STRA6* leads to syndromic microphthalmia 9 (MCOPS9/Matthew-Wood Syndrome – OMIM #601186), where severe systemic features include pulmonary, diaphragmatic and cardiac defects, resulting in death usually within the first 2 years of life.^2,217–219^ This phenotype is recapitulated by morpholino-induced knockdown, which exhibits microphthalmia, curved body axis, cardiac oedema and craniofacial defects due to disrupted retinoic acid signalling in the developing eye.^220^ A less severe phenotype was observed when an alternative morpholino was used where a small concentration of RNA was still detectable, indicating a dose-dependent mechanism which may explain the milder or isolated microphthalmia/coloboma phenotype (MCOPCB8) observed in some patients with homozygous *STRA6* mutations, including certain missense variants.^9,217^ Ocular and cardiac malformations in *stra6*-knockdowns were partially rescued by reduction of retinoic acid binding protein 4 (*rbp4*) using morpholino knockdown or 1-phenyl-2-thio-urea (PTU)-mediated inhibition (which downregulates *rbp4* mRNA expression at larval stages), demonstrating potential avenues for treatment *via* targeting of retinoic acid signalling pathways.^220–222^

#### Identification of novel variants through zebrafish studies

Where a new microphthalmia candidate gene or variant of unknown pathogenic significance is identified in a family through genetic investigation, zebrafish can be used to provide evidence of pathogenicity through expression studies in the early developing eye and through rapid gene knockdown in F0 fish and with validation of resulting phenotype. A novel association of *TMX3* with microphthalmia was validated with morpholinos targeting the *tmx3* zebrafish orthologue *zgc:110025*, resulting in significantly smaller eye size at 2 dpf.^17^ This phenotype was rescued by injection of human wildtype *TMX3* mRNA, but not by injection of the patient mutant mRNA (p.Arg39Gln), confirming a functional effect of the *TMX3* variant on eye growth.

Equally, phenotypic annotation of zebrafish knockdowns can be used to identify putative novel genes to screen unsolved patient cohorts. For example, *bco1* encodes a key enzyme for vitamin A formation and causes microphthalmia when knocked-down at the larval stage.^223^ Similarly, *rbm24a* has been found to positively control the mRNA stability of *sox2* transcripts, with gene knockdowns resulting in a small-eye phenotype.^224–227^ So far, no pathogenic mutations have been successfully detected in human orthologues *BCO1* or *RBM24* in microphthalmic patients, although disease-causing variants of *RBM24* are known to cause cardiomyopathy. Nevertheless, examining these genes for functional mutations in patients without a known genetic cause through next-generation sequencing could improve molecular diagnosis rates by broadening the mutational spectrum and inclusion in future panel-based diagnostic testing.^228^

#### Genetic modifiers in microphthalmia aetiology

Rapid generation of phenotypes in F0 fish provides an efficient method examine gene combinations to decipher epistatic interactions and oligogenic inheritance, aiding the investigation of multigenic factors in microphthalmia pathogenesis.^24^ Patients with pathogenic mutations in transcription factor *TFAP2A* display a variable ocular phenotype including microphthalmia, coloboma and cataract as part of branchio-oculo-facial syndrome (BOFS – OMIM #113620), but homozygous loss-of-function *tfap2a* zebrafish mutants and morpholino-induced knockdown display no ocular phenotype.^229,230^ Heterozygous mutations in *BMP4* cause syndromic microphthalmia 4 (OMIM #607932), but *bmp4*^−/−^ zebrafish have normal eye morphology. Transcription factor *tcf7l1a* plays a role in the Wnt signalling, but zebrafish *tcf7l1a*^−/+^ and *tcf7l1a*^−/−^ mutants do not have a disrupted ocular phenotype. However, injection of *tfap2a* morpholinos *tcf7l1a*^−/+^ and *tcf7l1a*^−/−^ mutants results in coloboma/anophthalmia, respectively, while injection into *bmp4*^−/−^ mutants causes microphthalmia and/or coloboma.^229^ *tcf7l1a* and *bmp4* variants sensitise the developing eye to the effects of additional deleterious mutations, implying human hypomorphic *TFAP2A* variants may contribute to developmental eye disorders when on a background with additional mutations, potentially explaining phenotypic variability. More severe microphthalmia has also been noted in *tfc7l1a*^−/−^ fish combined with mutations which when in isolation show no ocular phenotype (e.g. in *hesx1*) or a mild reduction in eye size (e.g. in *cct5* or *gdf6a*).^24,231^

Zebrafish studies show genetic interactions also influence syndromic heterogeneity, as *otx2* morpholino knockdowns display mild microphthalmia and shortening of the pharyngeal skeleton, but the combination of *otx2* and other otocephaly gene knockdowns (including *pgap1*, *prrx1* and *msx1*) result in more severe mandibular malformations, similar to craniofacial anomalies in patients with *OTX2-*associated otocephaly-dysgnathia complex.^39,172^ This work demonstrates that *otx2* interacts with other genetic loci to regulate development throughout the body, which may explain the high systemic variation observed in patients with *OTX2*-associated microphthalmia.^3,172^ Further investigation of multigenic factors in syndromic microphthalmia using zebrafish could clarify variability observed within families.

#### Modelling environmental causes of microphthalmia in zebrafish

Relatively few studies of environmental factors influencing eye growth have been performed; however, phenotypic variability observed within families indicates environmental factors could account for certain cases of variable penetrance and expressivity. Fertilisation and development of zebrafish *ex vivo* allows for easy modification of the embryonic environment. Vitamin A deprivation through pharmacological inhibition of enzyme retinaldehyde dehydrogenase in early wildtype zebrafish embryos results in a dose-dependent reduction in eye size, with high doses causing systemic features reminiscent of the MCOPS9 phenotype including cardiac oedema and mortality within the first days after treatment.^217,221^ Variable severity of MAC observed between siblings with retinoic signalling component *STRA6*, the molecular cause underlying MCOPS9, indicates environmental factors such as maternal retinoic acid intake may be the cause of clinical heterogeneity.^217^ Modelling these factors in zebrafish, where external conditions can be manipulated, can help explain the role of environmental factors in variable ocular and systemic phenotypes.

### Xenopus

#### Advantages and drawbacks of *Xenopus* models of ocular development

*Xenopus* have similar advantages as disease models to zebrafish, including external fertilisation and development and low cost.^232,233^ Unlike zebrafish, *Xenopus* are tetrapods, hence are evolutionarily more similar to humans, with *Xenopus tropicalis* sharing 79% of their genes with humans.^234–237^ *Xenopus* embryos are also larger in size, and able to tolerate extensive surgical manipulation, with transplantation of single cells to other parts of embryos in order to understand the role of interacting tissues and environments in development.^234^ However, like zebrafish, *Xenopus* genomes can contain duplicated genes, therefore clear understanding of compensation and subfunctionalisation is important when evaluating data.^238^ For example, genetic manipulation of *six6* in *Xenopus* shows diverged functionality of the duplicated genes, where knockdown of *six6.L* results in a more severe microphthalmia phenotype than knockdown of *six6.S.*^238^

Much of the early understanding of eye field specification, cell fate determination and the key regulators of oculogenesis were obtained from *Xenopus* studies.^33,233,239^ The development and mature eye structure is extremely similar between humans and *Xenopus*; nevertheless, the main difference is that *Xenopus*, like many amphibians and fish, can regenerate certain eye structures beyond embryogenesis. *Xenopus* have especially high capacity for ocular regeneration, and can produce new retinal cells through functional stem cell populations, and restore lost/damaged lens through transdifferentiation of the corneal epithelium.^46,233,240^ Overall, conservation of cellular and developmental processes, as well as genomic synteny with mammals, makes *Xenopus* a valuable resource for studying eye development and microphthalmia.^233,234,241^

#### Generation of microphthalmic *Xenopus*

Microphthalmic phenotypes can be generated in the F0 generation using morpholino-induced knockdowns or injection of genome editing tools at the single-cell stage, without the need for time-consuming crosses.^232,235,242,243^ For example, over 85% of TALENS-injected embryos to induce targeted gene disruption of *pax6a* and *pax6b* reveal perturbed eye formation and a spectrum of anophthalmia/microphthalmia phenotypes.^26,83,233,242^ Gain-of-function experiments have often been performed in *Xenopus* to understand molecular networks, as embryos tolerate injection with mRNA.^234^

#### Understanding molecular pathways in microphthalmia

Size, external development and regenerative properties of *Xenopus* embryos allows surgical manipulations to be performed which can provide new insights into the molecular pathways at the initiation of eye development. Early transplantation experiments were invaluable to establishing the timing of eye induction.^30,33^ Following this work, ectopic expression of EFTFs showed eye field initiation can only occur in the presence of *Otx2*, demonstrating a permissive role in regulating early eye development.^33,239^ Fluorescent tissue induced to express EFTFs and *Otx2* transplanted to different regions of host embryos form functional, organised eye-like structures, demonstrating the need for these factors alone to stimulate and coordinate oculogenesis.^27,244^ This understanding of the early regulators of eye development gleaned from *Xenopus* has been essential for extricating the molecular pathways underlying microphthalmia.

#### Modelling variable ocular and syndromic phenotypes

Developmental and genetic conservation with humans means *Xenopus* can be used to study both ocular and systemic phenotypes caused by microphthalmia-associated gene disruption. Overexpression of the epigenetic regulator *SMCHD1* through injection of wildtype or mutant mRNA results in craniofacial anomalies including microphthalmia, recapitulating the Bosma arhinia microphthalmia syndrome (BAMS – OMIM #603457) phenotype observed in patients with heterozygous missense mutations, confirming a gain-of-function mechanism.^245,246^ This phenotype is not recapitulated in mouse models, due to apparent redundancy of *Smchd1* function in rodents.^246^

Morpholino-induced knockdown of co-repressor gene *bcor* in *Xenopus* produces a microphthalmia phenotype, along with systemic features including skeletal and central nervous system abnormalities. These knockdowns phenotypically reflect *BCOR*-associated syndromic microphthalmia 2 (OMIM #300166), also known as oculofaciocardiodental syndrome as hallmarks include cataracts, microphthalmia, facial, cardiac and dental anomalies.^218,243,247,248^ Downregulation of transcription factor *Pitx2* in this model highlighted an upstream regulator role for *bcor*, demonstrating a shared pathway in *Xenopus* and humans, as heterozygous *PITX2* variants can cause anterior segment dysgenesis 4 (OMIM #137600) or Axenfeld–Rieger syndrome (OMIM #180500), where patients also exhibit dental hypoplasia and skeletal anomalies.^218^ Knockdown of *bcor* in zebrafish does not produce a small eye, instead displaying a less severe ocular coloboma phenotype, and no ocular phenotype has been observed in mouse models of *Bcor*.

#### Identification of novel variants through *Xenopus* studies

Rapid ocular development, along with tolerance for genetic manipulation and injection of additional genetic material, means genes suspected to be involved in microphthalmia pathogenesis can be easily assessed in *Xenopus* using morpholino-induced knockdowns or overexpression to evaluate hypermorphic variants. MicroRNAs (miRNAs) are post-transcriptional regulators of gene expression.^249^ While not currently associated with microphthalmia, their role in eye development and disease is being revealed.^249,250^ Targeted knockdown or overexpression of *miR-199* in *Xenopus* results in small eyes and reduced cell proliferation in the eye field due to disruption of EFTF expression including *rax1.*^251^ This phenotype is rescued by blocking the *miR-199* binding site, demonstrating a distinct role of miRNAs in eye development and ocular maldevelopment, and a novel set of targets for drug treatments. Additional candidates for patient screens originating from *Xenopus* overexpression modelling include *siah-2*,^252,253^ *E*-*NTPDase*,^254^ *PNAS-4*^255^ and *pparγ*^256^ and knockdowns of *sdr16c5*,^257^ *frs3*^258^ and *psf2.*^259^ Although none of the candidates listed have yet been identified in microphthalmic cohorts, as frequency of next-generation sequencing escalates and large databases such as from the 100,000 genomes project can be analysed in more depth, there is increased capability to identify novel genes in patients through screening performed in animal models.^260^

#### Modelling environmental causes of microphthalmia in *Xenopus*

External development of *Xenopus* embryos allows for evaluation of adverse effects of environmental changes on ocular development. Alcohol consumption during pregnancy can cause Foetal Alcohol Spectrum Disorder (FASD), leading to microphthalmia, short stature, microcephaly and facial anomalies. Exposure of *Xenopus* embryos to ethanol between the late blastula and early/mid gastrula stages (stage 8.5–18) recapitulates phenotypic aspects of FASD, including shortened rostro–caudal axis, microcephaly and microphthalmia, due to antagonism of vital retinoic acid signalling pathways through competitive inhibition.^261^ This knowledge could be beneficial for understanding how genetic and environmental interaction impact eye development and help explain clinical heterogeneity in microphthalmic cohorts.

## Human cellular models of microphthalmia

As discussed, differences exist in genetic regulation and disease manifestation between humans and animals. Hence, *in vitro* human cellular disease models can overcome species-dependent variation for studying molecular mechanisms and therapeutic compound testing, while also reducing the use of animal experimentation.

### Generation and advantages/drawbacks of different types of cellular models

#### Primary cell lines

Cells derived directly from patients with molecularly confirmed cause allow researchers to study how specific variants disrupt human cell function, and thereby investigate genotype–phenotype correlations from a molecular and cellular perspective. Furthermore, developing and testing the effects of drugs on patient-derived cells increases capacity to determine drug efficacy, creating more reliable data for which treatments might be successful in clinical trials as well as potential for more personalised medicine options.^262–265^ However, a drawback of primary cell lines is as they senesce, they display changes in function and morphology, and eventually stop replicating; for example, primary RPE cells cannot be passaged more than 4–6 times.^266^ Additionally, developmental cell types relevant to the onset of microphthalmia such as retinal progenitor cells cannot be derived from adult tissue, and consequently must be isolated from embryonic tissues, which are in short supply and have ethical implications surrounding their usage.^267,268^

#### Immortalised cell lines

Immortalised cell lines provide an unlimited supply of cells at a relatively low cost and are easy to maintain.^269^ They are useful for studying various molecular functions in cellular processes, as they are generally tolerant of transfection with exogenous genetic material, and so can be induced to express genes of interest, enabling investigation into their mechanism of action in health and disease. However, misidentification and contamination remain widespread problems in producing reliable data from cell lines.^270,271^ Moreover, due to genetic manipulation required to produce the immortalised line, cells may no longer represent their cell type of origin, such as the epithelial phenotype of ARPE-19 cells which diminishes within 3–4 passages, partially due to loss of key claudin tight junctions resulting in reduced functionality.^272,273^

#### Embryonic stem cells (ESCs)/Human induced pluripotent stem cells (hiPSCs)

Embryonic stem cells (ESCs) and human induced pluripotent stem cells (hiPSCs) have the capacity to differentiate into any lineage, and therefore can model cellular functions and molecular regulation in any cell type, at different stages of development.^262,274–276^ By providing an unlimited source of cells for disease modelling, ESCs/hiPSCs are an excellent resource for research and developing therapies, although can be expensive and more difficult to culture than other cells.^277^ HiPSCs are also a promising source of cells to treat disease by transplanting into patients, either as differentiated cells, or in a pluripotent/multipotent state.^278,279^ Cell-based therapies are being developed for multiple ocular diseases, such as age-related macular degeneration and retinitis pigmentosa, and show initial success with many ongoing clinical trials.^278–280^ The majority of ocular cell therapies currently focus on degenerative diseases, but transplantation of stem/progenitor cells may yet prove valuable for treating developmental disorders such as microphthalmia, by boosting eye growth postnatally.^281,282^

#### 3D cellular models

Traditionally, cells are grown as a monolayer of a specific cell type on a flat surface. However, 2D cell culture has been shown to alter cell morphology, gene expression and function.^283–285^ Furthermore, monoculture of a single cell type lacks the cross-cell-type signalling necessary to recapitulate the *in vivo* complexity.^286,287^ Recreating the natural environment experienced in the developing eye using 3D culture techniques with multiple interacting cell types facilitates collection of more clinically relevant data.^264,288^ Organoids mimic development through restricted division of progenitor cells and expression of distinct cellular adhesion molecules which allow temporal and spatial organisation of multiple cell types, in a manner similar to that of organs.^288^ As such, organoids allow study of human organogenesis at developmental stages which would be otherwise inaccessible, such as within the first weeks of pregnancy.

Optic cup-like organoids were first generated by the Sasai group, using mESCs in 2011, then human ESCs in 2012.^289–292^ Their work showed self-organisation of cells into distinct layers reflecting the NR and RPE of the developing optic cup, although with inconsistent efficiency in forming stratified retina, which may have been the result of missing surface ectodermal signalling molecules from the presumptive lens.^35,291,293,294^ Modifications (such as addition of retinoic acid receptor antagonist AGN193109 at early stages to improve yield of cells expressing *Rax*^295^) have led to numerous protocols shown to recapitulate stages of early embryonic eye development using transcriptomic analysis.^296–308^

One major criticism of organoids is the heterogeneity in differentiation efficiency observed within and between cell lines, partially due to differences in endogenous genetics and epigenetics.^293,309–317^ Attempts to combat background genetic/epigenetic variability include creation of isogenic lines through CRISPR/Cas9 gene editing to induce/correct patient mutations, to reduce noise and generate more reliable data.^318,319^ It should also be noted that lack of additional signals such as the embryonic axis means organoid structures are often highly heterogeneous, with random relative positioning of tissue regions, such as RPE and NR in retinal organoids.^288^ Additionally, current constraints of *in vitro* organoid modelling include lack of vasculature thus poor nutrient diffusion, and absent surrounding tissues, which may result in loss of vital external developmental cues.^35,288,294^ However, advances in co-culturing techniques and organ-on-a-chip technologies may provide a solution for more complex cellular modelling, by facilitating signalling between different cell types, and a more vasculature-like perfusion of nutrients across organoids.^286,320,321^ The potential of these more advanced culturing systems for drug toxicity screening has been demonstrated through chloroquine and gentamicin treatment to induce retinopathies, although successful use of retinal organoids in drug discovery screens has yet to be reported.^320,322^

#### Understanding molecular pathways in microphthalmia

Studying the genetic basis of microphthalmia directly in human cells has a clear advantage over animal models, as genetic pathways and potential therapies can be studied without possibility for divergence or functional redundancy. By modelling gene function at a cellular level in human tissue, a more translational understanding of microphthalmia pathogenesis can be established. Homozygous frameshift mutations in *FAT1* have been associated with colobomatous microphthalmia, ptosis, syndactyly and facial dysmorphism in patients.^323^ Study of RPE cells showed FAT1 localised to cell–cell junctions required for optic fissure fusion in eye development, and knockdown of *FAT1* using short hairpin RNA (shRNA) resulted in disruption of β-Catenin, ZO-1 and F-actin fibres at junction sites, and a failure of RPE cells to form an organised epithelial monolayer.^323^ These disruptions were not observed in differentiating RPE tissue from *in vivo Fat*^−/−^ mouse models, although mice did display a microphthalmia and coloboma phenotype. The ability to study molecular pathways in human cells allows for clarity in where molecular mechanisms are conserved and where they deviate from animal models.

In 2014, Phillips *et al.* generated optic vesicle-like models of early eye development with iPSCs derived from a microphthalmic patient with a homozygous *VSX2*^R200Q^ mutation.^324^ Molecular techniques including RNAseq and ChIPseq identified that WNT pathway components were direct targets for *VSX2* DNA binding and transcriptional downregulation in retina development. Upregulation of the WNT pathway in the *VSX2*-disrupted models resulted in erroneous RPE differentiation, partially rescued by pharmacological inhibition of the WNT pathway.^29,324^ Furthermore, supplementation with growth factors including FGF9 partially rescued the phenotype in mutant organoids, although suppression of FGF9 alone in wildtype organoids did not produce a phenotype, indicating redundancy of pathways in retinal development.^325^ The valuable insights gained from this study demonstrate the ability of these 3D cellular models to advance our understanding of how individual genes function in human eye development and which pathways are disrupted in microphthalmic patients with the corresponding variant.

#### Modelling variable ocular and syndromic phenotypes

Studying the impact of disease-causing proteins on cell function can elucidate the effect of different alleles and genetic/epigenetic background on phenotypic variability. *FZD5* is a transmembrane receptor which regulates WNT signalling in the early optic vesicle.^326^ Investigation of *FZD5* in HEK (human embryonic kidney) cells revealed that transfection of microphthalmic patient-originated cDNA produced truncated protein which did not localise to the outer cell membrane or mediate WNT signalling like wildtype protein, instead inhibiting the pathway due to antagonistic competition, resulting in a dominant-negative effect.^326^ Heterozygous pathogenic mutations in *FZD5* have predominantly been identified in coloboma cohorts, but in one large family with the frameshift variant c.656delCinsAG, p.Ala219Glufs*49, two members were non-penetrant.^326,327^ Animal models also display variable MAC phenotypes, such as zebrafish with *fzd5* knockdown or overexpression of mutant protein,^326^ and *Fzd5*^−/−^ mice which exhibit 50% penetrance of mild microphthalmia/coloboma.^328^ This may be the result of overlapping function with *Fzd8*, as triallelic *Fzd5*^−/−^;*Fzd8*^+/−^ mutants develop severe retinal coloboma and microphthalmia with full penetrance. No *FZD8* protective alleles were identified in non-penetrant individuals from whole exome sequencing; however, further analysis of human cellular models could help identify other effects of genetic background or compensatory gene mechanisms on *FZD5* function.

Cell models are not representative of the whole organism and hence it is more difficult to explore systemic manifestations. However, they can be used to extrapolate tissue involvement through investigating the transcriptome and molecular pathways, as gene ontology tools can link developmental pathways in other parts of the body to shed light on syndromic features. For example, transcriptomic analysis of zebrafish optic fissure tissue identified differentially expressed genes between optic fissure and dorsal retina which are known to be involved in heart development (*tbx2a*/*3a*), providing new pathways to explore through cellular research.^329^ In addition, patient-derived fibroblasts with a heterozygous splice-site *NAA10* variant show reduced cell proliferation and disrupted retinoic acid signalling, which may explain both microphthalmia and extraocular growth defects observed in patients with syndromic microphthalmia 1 (Lenz microphthalmia syndrome – OMIM #309800).^330^

#### Identification of novel variants with cellular models

Converting genomic annotations from animal models to humans can be misleading, due to divergence in genetic regulation of eye development. Evidence from human cellular studies can therefore be more practical for identifying and validating novel candidates. Generation of transcriptomic and epigenomic data from human-derived 3D microphthalmic models could provide datasets from which pathway components and disease mechanisms can be identified, providing both validation for putative genetic causes found in patients as well as resources to discover new genes to screen in microphthalmic cohorts by next-generation sequencing. To date, few 3D cellular disease models have been generated, but as protocols grow more efficient, and multi-omic technologies become more affordable, cellular modelling could become an effective strategy for detecting molecular causes of microphthalmia.

#### Modelling environmental causes of microphthalmia in cells

The effect of exogenous chemicals on cellular function can be quickly investigated in 2D cell culture, due to efficient diffusion of compounds. Retinoic acid treatment of ARPE-19 cells induced dose-dependent increase in *RAR*β mRNA and protein within 24 h, which was inhibited by treatment with antagonist LE135.^331^ Utilising more complex 3D models, toxins and potential treatments can be applied directly to mature human ocular tissues without bioavailability and drug metabolism issues, allowing greater understanding of effect on ocular development and its regulation. Importantly, using patient-derived cells can shed light on the effects of environmental factors on different genetic backgrounds and particular modifiers, leading to more precise clinical advice and care.

## Conclusion

Through work studying patients, animals and cellular models, considerable progress has been made in understanding the genetic basis of eye development, and how dysregulation of molecular pathways can result in microphthalmia. Over 90 monogenic causes of microphthalmia have been identified, and yet molecular diagnosis can only be made in less than 10% of unilateral patients and few genotype–phenotype correlations have been established. Numerous animal models for microphthalmia have been generated; however, many still have not been genetically characterised (including 25% of mouse lines) and several causative microphthalmia genes have not been disrupted in animals.^25^ Many known human genetic variants have not been studied in detail due to a lack of a corresponding model. Screening animal lines for novel candidate genes/genetic modifiers and functionally validating variants identified in patients could increase understanding of the roles of disease-causing genes, improve molecular diagnostic rates and provide patients with appropriate multidisciplinary care and genetic counselling by clarifying genotype–phenotype relationships.

Cutting-edge developments in 3D cellular modelling techniques show potential as an animal-free approach for deepening understanding of human eye development and molecular disease mechanisms at early stages of oculogenesis, which would otherwise be inaccessible to study, as well as providing promising results in understanding patient-specific mutations and developing novel therapeutics.^29,264^ Nevertheless, whole-organism modelling in animals is necessary for understanding the systemic effect of gene disruption and screening drugs, particularly when studying syndromic microphthalmia.^155^ Research on a combination of animal and cellular models is essential to gaining a clear picture of the molecular basis of microphthalmia and developing life-changing treatments.

## Supplemental Material

sj-xlsx-1-trd-10.1177_2633004021997447 – Supplemental material for Animal and cellular models of microphthalmia Click here for additional data file. Supplemental material, sj-xlsx-1-trd-10.1177_2633004021997447 for Animal and cellular models of microphthalmia by Philippa Harding, Dulce Lima Cunha and Mariya Moosajee in Therapeutic Advances in Rare Disease
